# Prognostic value of routine blood biomarkers in 3-year survival of resectable colorectal cancer patients: a prognostic nomogram for clinical practice

**DOI:** 10.1007/s00384-025-04848-3

**Published:** 2025-03-05

**Authors:** David Moro-Valdezate, José Martín-Arévalo, Coral Cózar-Lozano, Stephanie García-Botello, Leticia Pérez-Santiago, David Casado-Rodrigo, Carolina Martínez-Ciarpaglini, Noelia Tarazona, Vicente Pla-Martí

**Affiliations:** 1 Department of Surgery, University of Valencia, Av. Blasco Ibáñez, 17, 46010 Valencia, Spain; 2 Colorectal Surgery Unit, Department of General and Digestive Surgery, INCLIVA Biomedical Research Institute, Hospital Clínico Universitario de Valencia, Av. Blasco Ibáñez, 17, 46010 Valencia, Spain; 3 Department of Pathology, INCLIVA Biomedical Research Institute, University of Valencia, Valencia. CIBERONC, Instituto de Salud Carlos III, Madrid, Spain; 4 Department of Medical Oncology, INCLIVA Biomedical Research Institute, University of Valencia, Valencia. CIBERONC, Instituto de Salud Carlos III, Madrid, Spain

**Keywords:** Blood biomarkers, Colorectal cancer, Prognosis

## Abstract

**Purpose:**

This study aimed to develop a prognostic model for colorectal cancer (CRC) patients using biomarkers from routine preoperative peripheral blood examinations combined with clinical factors.

**Methods:**

This observational study comprised CRC patients (stages I–III) who underwent curative surgery between January 2011 and December 2019. Study variables included patient demographics, tumour characteristics, and immune/inflammatory markers from preoperative blood tests. Cut-off thresholds for continuous variables were determined using maximally selected rank statistics. Univariate and multivariate analyses identified variables associated with 3-year cancer-specific survival (CSS) and disease-free survival (DFS). Cox regression models were developed and validated using a random split-sample approach. Nomograms based on these models were constructed, and receiver operating characteristic (ROC) curves were generated for 12, 24 and 36 months.

**Results:**

A total of 764 patients were included. Independent factors for 3-year DFS included laparoscopic surgery, prognostic nutritional index (PNI), neutrophil count, lymphocyte count, and Charlson comorbidity index. The DFS prediction model showed AUC values of 66.6%, 64.8%, and 69% for years 1, 2, and 3, respectively. For CSS, independent factors included age, systemic immune-inflammation index (SII), serum albumin, and platelet count, with AUC values of 89.2%, 76.8%, and 71% for years 1, 2, and 3. The most significant contributors to the CSS model were SII and platelet cut-off values.

**Conclusion:**

Inflammatory biomarkers combined with clinical parameters robustly predict 3-year survival outcomes in CRC patients undergoing curative resection. These findings highlight the importance of systemic inflammation in CRC prognosis and support its inclusion in preoperative risk stratification.

## Introduction

One of the most frequent questions posed by patients newly diagnosed with colorectal cancer (CRC) concerns their prognosis. Providing an accurate preoperative prognosis remains challenging due to limitations in existing staging methods. While computed tomography (CT) is routinely used for preoperative staging, its sensitivity is suboptimal, particularly for early-stage CRC [1–3]. This highlights the need for supplementary tools to improve staging accuracy.

Systemic inflammation is increasingly recognized as a key factor in CRC progression. Inflammatory mediators promote tumour progression by increasing vascular permeability, facilitating cancer cell infiltration, contributing to tumour cell adhesion to the endothelium, and enhancing stromal invasion [4]. Recent studies have demonstrated that inflammation-based markers derived from routine blood tests—such as neutrophil, lymphocyte, monocyte, and platelet counts—can predict outcomes in CRC patients [5, 6]. Ratios between these leukocyte populations, including the neutrophil-to-lymphocyte ratio (NLR), derived neutrophil–lymphocyte ratio (DNLR), monocyte-lymphocyte ratio (MLR), and platelet-to-lymphocyte ratio (PLR), have also been linked to survival outcomes [7–11].

However, despite the increasing recognition of these inflammatory indices as prognostic factors, their predictive performance remains inconsistent across different clinical settings. While some studies report strong associations with survival outcomes, others suggest that their prognostic ability may be influenced by tumour biology, patient comorbidities, or treatment modalities [9, 12]. Additionally, discrepancies in the optimal cut-off values across studies have hindered the universal application of these biomarkers in clinical practice [13]. To overcome these limitations, the present study integrates multiple inflammatory and nutritional parameters with clinical factors, aiming to enhance the predictive accuracy of the prognostic model.

Novel scoring systems, such as the prognostic nutritional index (PNI) and the systemic immune-inflammation index (SII), have shown promise in predicting both overall survival and disease-free survival (DFS) [7, 14–16]. This study aimed to develop a prognostic model integrating these biomarkers with clinical parameters to improve preoperative risk assessment and guide treatment decisions for CRC patients. By assessing the predictive accuracy of these biomarkers, our study aims to enhance the understanding of their clinical utility in preoperative risk stratification.

## Material and methods

### Study design and population

This observational study was conducted from January 2011 to December 2019 at the Colorectal Surgery Unit of the University Clinical Hospital of Valencia, Spain. Eligible patients were over 18 years old and underwent elective curative surgery for preoperative clinical stage I-III CRC, with a minimum follow-up of 36 months. Exclusion criteria included need for neoadjuvant therapy, non-adenocarcinoma histology, postoperative death within 30 days, transanal minimally invasive surgery, palliative procedures, and incomplete follow-up. The study adhered to STROBE guidelines.

### Study endpoints

The primary endpoints were 3-year cancer-specific survival (CSS) and disease-free survival (DFS). Disease recurrence was defined as the reappearance of neoplastic cells at the primary site or distant locations. Cancer-specific mortality referred to deaths directly attributable to CRC progression.

### Data collection

Patient data were retrieved from a prospectively maintained institutional database. All surgical procedures were performed by specialized colorectal surgeons. Variables included demographics, American Society of Anaesthesiologists (ASA) score, Charlson comorbidity index, tumour TNM staging (American Joint Committee on Cancer, 8th edition) [17], tumour location (categorized as ascending colon, transverse colon, splenic flexure, descending colon, sigmoid colon, or rectum), and histological differentiation grade. Preoperative blood samples were collected within one month prior to surgery and were analysed for neutrophils, lymphocytes, monocytes, platelets, haemoglobin, and serum albumin. Calculated indices included neutrophil–lymphocyte ratio (NLR), derived neutrophil–lymphocyte ratio (DNLR), monocyte-lymphocyte ratio (MLR), platelet-lymphocyte ratio (PLR), systemic immune-inflammation index (SII), and prognostic nutritional index (PNI). SII was calculated as (neutrophil count × platelet count) / lymphocyte count, and PNI as (serum albumin [g/dL] × 10) + (lymphocyte count × 0.005).

### Patient follow-up

Follow-up care was conducted jointly by the Colorectal Surgery and Medical Oncology departments according to the institution’s post-operative protocol. Serum carcinoembryonic antigen (CEA) levels were measured every three months during the first postoperative year, biannually during the second and third years, and annually thereafter. Annual chest and abdominal CT scans were performed or scheduled when elevated CEA levels were detected. Colonoscopy was conducted one year after surgery, with subsequent screenings every 3–4 years, or as indicated by clinical suspicion of local recurrence. The minimum follow-up period for study inclusion was 36 months.

### Ethics approval and consent to participate

The study protocol was reviewed and approved by the local ethics committee. Due to its observational nature, informed consent for study participation was waived, in accordance with institutional policies. Data analyses were conducted using anonymized clinical records to maintain patient confidentiality.

### Statistical analysis

Data preprocessing and quality control were performed by the first two authors, ensuring data accuracy and consistency. This process involved cross-referencing patient records, addressing missing or inconsistent values, and eliminating unreliable entries. Descriptive analyses were conducted for all study variables. Normality was assessed using Q-Q plots and the Shapiro–Wilk test. Continuous variables were presented as mean ± standard deviation or median (range) based on their distribution, while categorical variables were described as frequencies and percentages.

For survival analysis, continuous variables were discretized using the maximally selected rank statistics method, which identifies cut-off points that maximize survival function differences between groups. Survival models were then compared using maximum likelihood estimation to optimize these thresholds. Univariate analysis identified variables associated with 3-year DFS and CSS. These significant variables were subsequently incorporated into a multivariate Cox proportional hazards regression model for 3-year DFS and CSS. The 3-year time frame was chosen based on prior evidence suggesting that machine learning-based predictive models tend to achieve optimal performance at this interval, likely due to the increasing influence of post-treatment factors over longer follow-up periods [18]. A forward stepwise approach was applied to select significant predictors, while multicollinearity was assessed using the variance inflation factor (VIF). Variables with high VIF values (VIF ≥ 4), indicating potential collinearity, were evaluated, and redundant predictors were excluded as needed to enhance model stability.

Internal validation of the prognostic models was performed using a 70–30 split-sample method, with 70% of cases randomly allocated to a training set and 30% to a test set. Nomograms based on Cox regression models were developed for predicting 3-year CSS and DFS. Model calibration was assessed using bootstrap resampling, while predictive accuracy over time was evaluated through time-dependent receiver operating characteristic (ROC) curves at 12, 24, and 36 months. Statistical significance was set at *p* ≤ 0.05. Data analyses were conducted using R version 4.3.0 (R Foundation for Statistical Computing, Vienna, Austria) on Windows 11.

## Results

**Fig. 1:**
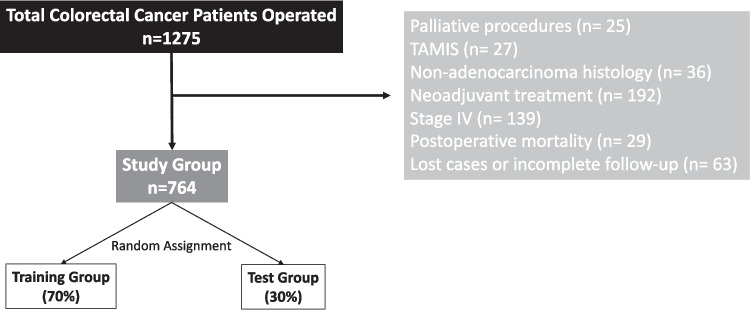
Study flowchart illustrating patient selection, exclusion criteria, and random allocation into training and test groups for model development

From a cohort of 1,275 patients diagnosed with CRC who underwent elective surgery during the study period, a total of 764 patients met the inclusion criteria (Fig. 1). The median follow-up of patients was 72 (range = 134) months. Baseline patient demographics, preoperative laboratory parameters, and tumour- and surgery-related characteristics are summarized in Table 1. The cohort comprised 60.9% males, with a median age of 65 years. Most patients were classified as ASA II (45.5%) or ASA III (49.3%). The median Charlson comorbidity index score was 5 (range = 13). The most common tumour location was the ascending colon (36.4%), and the laparoscopic approach was the predominant surgical technique (74.6%). Most tumours were low-grade differentiated (94.9%), and stage II was the most frequently observed TNM classification (38.7%).

**Table 1:** Baseline patient characteristics, preoperative laboratory test outcomes, tumour and surgery features

Patient variables	
Age (years)	73 (72)
Sex (female)	299 (39.10%)
ASA	
I	34 (4.50%)
II	348 (45.50%)
III	377 (49.30%)
IV	5 (0.70%)
Charlson comorbidity index	5 (13)
Body mass index	27.68 (32.97)
Preoperative laboratory test variables	
Total leucocyte (10^9^ / L)	7.9 (21.70)
Neutrophil (10^9^ / L)	4.88 (21.05)
Lymphocyte (10^9^ / L)	1.61 (3.93)
Monocyte (10^9^ / L)	0.64 (3.18)
Platelet (10^9^ / L)	254 (426)
Neutrophil / Lymphocyte ratio	2.51 (24.95)
Derived neutrophil / Lymphocyte ratio	1.5 (1.84)
Lymphocyte / Monocyte ratio	2.94 (6.33)
Platelet / Lymphocyte ratio	139.49 (722.93)
Immuno-inflammatory index	573.71 (11,685.62)
Haemoglobin (g/dl)	12.5 (11.60)
Seric albumin (g/dl)	4.1 (3)
Nutritional prognostic index	39.01 (26)
Tumour variables	
Tumour location	
Ascendant colon	278 (36.40%)
Transverse colon	48 (6.30%)
Splenic flexure of the colon	35 (4.60%)
Descendant colon	30 (3.90%)
Sigmoid colon	203 (26.60%)
Upper third of the rectum	97 (12.70%)
Middle third of the rectum	43 (5.60%)
Lower third of the rectum	30 (3.90%)
Tumour differentiation grade	
High Grade	39 (5.10%)
Low Grade	725 (94.90%)
Tumour stage (TNM – AJCC, 2018)	
0	30 (3.90%)
I	195 (25.50%)
II	296 (38.70%)
III	243 (31.80%)
Surgery variables	
Operative time (minutes)	135 (402)
Surgical approach (Laparoscopy)	570 (74.60%)
Surgical procedure	
Right colectomy	300 (39.30%)
Segmental splenic flexure colectomy	17 (2.20%)
Left colectomy	48 (6.30%)
Sigmoid resection	173 (22.60%)
Hartmann’s procedure	23 (3%)
Low anterior resection of rectum	151 (19.8%)
Abdomino-perineal resection	12 (1.60%)
Total Colectomy	18 (2.40%)
Subtotal Colectomy	22 (2.90%)
Preoperative transfusion	62 (8.10%)

*ASA* American society of anaesthesiologists

*AJCC* American joint committee on cancer, 8th edition (2018)

Statistics presented as median (range) or *n* (%)

Several preoperative laboratory parameters were significantly associated with tumour differentiation grade. These included serum albumin (*p* = *0.008*), haemoglobin (*p* = *0.001*), neutrophils (*p* < *0.001*), platelets (*p* < *0.001*), total leukocytes (*p* < *0.001*), monocytes (*p* = *0.002*), NLR (*p* < *0.001*), MLR (*p* = *0.001*), PLR (*p* = *0.001*), (*p* = *0.009*), SII (*p* < *0.001*), and PNI (*p* = *0.008*).

Significant associations were also observed between study variables and tumour stage, including serum haemoglobin level (*p* < *0.001*), serum albumin (*p* = *0.004*), neutrophils (*p* = *0.037*), platelets (*p* < *0.001*), PLR (*p* < *0.001*), DNLR (*p* = *0.038*), SII (*p* < *0.001*), PNI (*p* = *0.005*), and age (*p* = *0.039*). Optimal cut-off thresholds for continuous variables were determined based on their relationship with DFS and CSS times (Table 2).

**Table 2:** Cut-off points for study variables based on 3-year disease-free survival and 3-year cancer-specific survival from oncologic disease progression

	3-year disease-free survival		3-year cancer-specific survival	
Study variables	Cut point	Statistic	Cut point	Statistic
Total leucocyte *	10.04	2.89	10.54	3.01
Neutrophils *	6.97	3.42	7.30	3.52
Lymphocytes *	1.17	2.95	1.69	2.04
Monocytes *	0.75	1.44	0.91	2.53
Platelets *	177.00	3.17	371.00	5.02
NLR	3.06	3.82	3.42	4.09
DNLR	1.35	2.32	1.31	2.63
MLR	1.73	1.40	3.33	2.93
PLR	150.83	3.12	221.74	3.97
Haemoglobin (g/dl)	14.60	2.21	10.60	4.48
Albumin (g/dl)	4.00	2.90	3.60	5.20
PNI	40.01	3.14	36.01	5.25
SII	919.48	3.57	1401.01	5.50
Age	73.00	4.04	79.00	3.82
Charlson comorbidity index	5.00	4.22	6.00	1.89
BMI	30.84	1.68	24.11	3.31

*Haematic cells count

*NLR* Neutrophil–lymphocyte ratio, *DNLR* Derived neutrophil–lymphocyte ratio, *MLR* Monocyte-lymphocyte ratio, *PLR* Platelet-lymphocyte ratio, *PNI* Prognostic nutritional index, *SII* Systemic immune-inflammation index, *BMI* Body mass index

Univariate analysis identified variables significantly associated with DFS (Table 3), including cut-off thresholds for leukocyte count (HR: 0.36, 95% CI: 0.17–0.77, *p* = *0.009*), neutrophils (HR: 0.13, 95% CI: 0.03–0.51, *p* = *0.004*), lymphocytes (HR: 4.8, 95% CI: 1.5–15, *p* = *0.007*), albumin level (HR: 0.66, 95% CI: 0.47–0.93, *p* = *0.018*), PNI (HR: 0.62, 95% CI: 0.43–0.87, *p* = *0.006*), SII (HR: 0.4, 95% CI: 0.23–0.69, *p* = *0.001*), NLR (HR: 0.46, 95% CI: 0.29–0.73, *p* = *0.001*), PLR (HR: 0.6, 95% CI: 0.41–0.89, *p* = *0.001*), and Charlson comorbidity index (HR: 1.8, 95% CI: 1.3–2.5, *p* = *0.001*). The laparoscopic approach was associated with improved DFS (HR: 0.64, 95% CI: 0.44–0.9, *p* = *0.02*). However, sex (*p* = *0.49*) and tumour location (colon vs. rectum, *p* = *0.51*) did not significantly influence 3-year DFS. For 3-year cancer-specific mortality, the only variables not associated with survival in univariate analysis were lymphocyte count threshold and tumour location (Table 3). The laparoscopic approach was significantly associated with lower mortality risk from disease progression at 3 years (HR: 0.46, 95% CI: 0.26–0.82, *p* = *0.008*).

**Table 3:** Univariate analysis for 3-year disease-free survival and 3-year cancer-specific survival from oncologic disease progression

	3-year disease-free survival			3-year cancer-specific survival		
	β	HR (95% CI)	*p*-value	β	HR (95% CI)	*p*-value
Total leucocyte	−1.00	0.36 (0.17 – 0.77)	***0.009***	1.00	2.90 (1.50 – 5.60)	***0.003***
Neutrophils	−2.10	0.13 (0.03 – 0.51)	***0.004***	1.20	3.30 (1.70 – 6.30)	** < *0.001***
Lymphocytes	1.60	4.80 (1.50 – 15.00)	***0.007***	−0.54	0.58 (0.33 – 1.00)	0.060
Monocytes	0.28	1.30 (0.92 – 1.90)	0.130	0.88	2.40 (1.20 – 4.70)	***0.010***
Platelets	−0.42	0.66 (0.43 – 1.00)	0.055	1.50	4.40 (2.40 – 7.90)	** < *0.001***
NLR	−0.78	0.46 (0.29 – 0.73)	***0.001***	1.20	3.20 (1.80 – 5.60)	** < *0.001***
DNLR	0.45	1.60 (0.98 – 2.50)	0.063	2.10	8.10 (1.20 – 59.00)	***0.038***
MLR	0.60	1.80 (0.85 – 3.90)	0.120	−0.93	0.39 (0.20 – 0.77)	***0.007***
PLR	−0.51	0.60 (0.41 – 0.89)	***0.001***	1.20	3.40 (1.90 – 6.30)	** < *0.001***
Haemoglobin (g/dl)	−0.44	0.64 (0.38 – 1.10)	0.110	−1.20	0.30 (0.17 −0.53)	** < *0.001***
Albumin (g/dl)	−0.42	0.66 (0.47 – 0.93)	***0.018***	−1.50	0.23 (0.13 – 0.41)	** < *0.001***
PNI	−0.49	0.62 (0.43 – 0.87)	***0.006***	−1.30	0.28 (0.15 – 0.52)	** < *0.001***
SII	−0.93	0.40 (0.23 – 0.69)	***0.001***	1.60	4.90 (2.90 – 9.10)	** < *0.001***
Age	0.54	1.70 (1.20 – 2.40)	***0.007***	1.00	2.80 (1.60 – 5.00)	** < *0.001***
BMI	−0.26	0.77 (0.53 – 1.10)	0.170	−0.78	0.47 (0.24 – 0.87)	***0.017***
Charlson’s index	0.58	1.80 (1.30 – 2.50)	***0.001***	0.60	1.80 (1.00 – 3.20)	***0.042***

The cut-off point of each variable was used for the univariate analysis

*NLR* Neutrophil–lymphocyte ratio, *DNLR* Derived neutrophil–lymphocyte ratio, *MLR* Monocyte-lymphocyte ratio, *PLR* Platelet-lymphocyte ratio, *PNI* Prognostic nutritional index, *SII* Systemic immune-inflammation index, *BMI* Body mass index

**Fig. 2:**
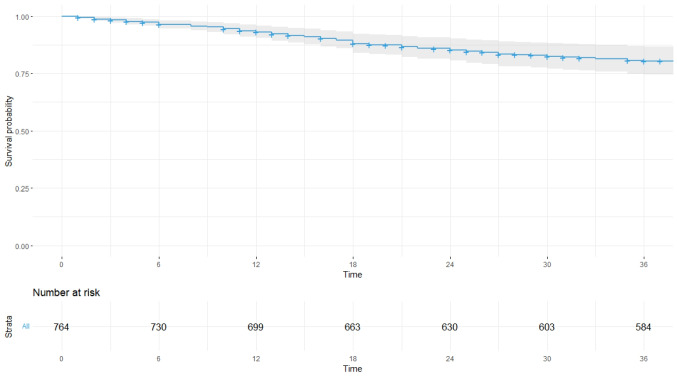
Kaplan–Meier curves showing 3-year disease-free survival probabilities based on the Cox regression model

**Fig. 3:**
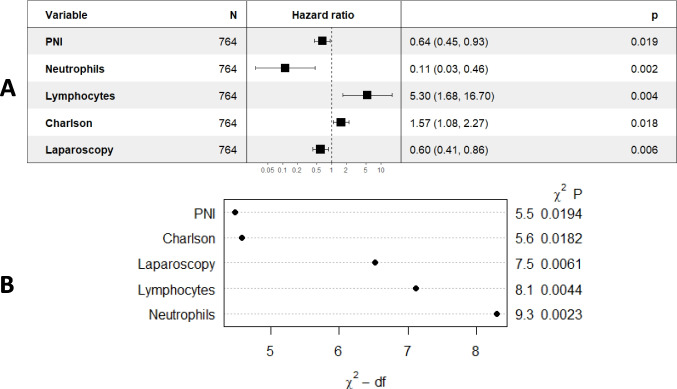
**A** Forest plot of the Cox regression-based model for 3-year disease-free survival. The plot shows the confidence intervals and relative risks for each variable included in the model. **B** Graphical representation of variable importance in the model for 3-year disease-free survival, calculated through Cox regression analysis. (PNI: Prognostic Nutritional Index)

**Fig. 4:**
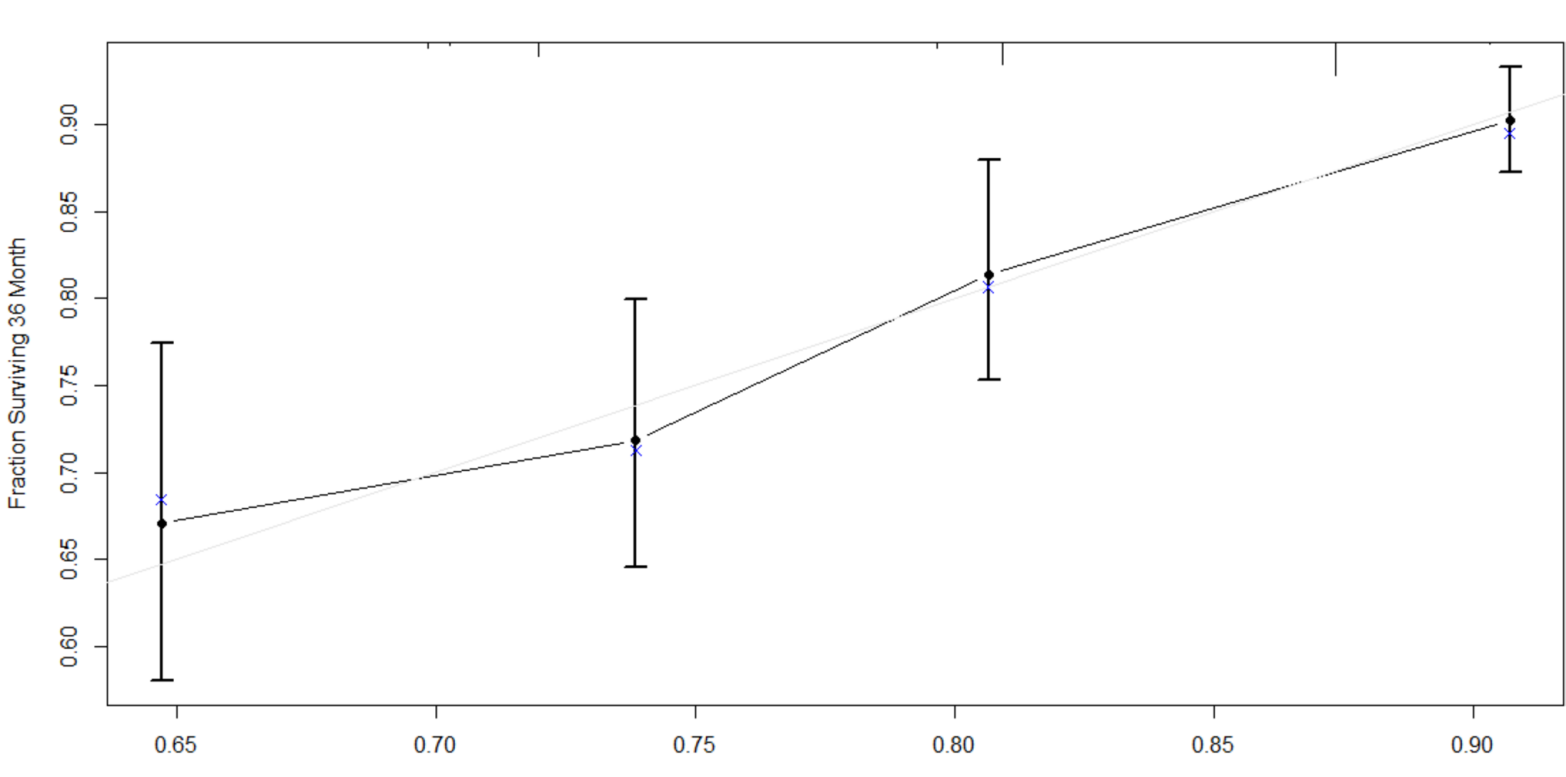
Calibration plot of Cox regression-based model for 3-year disease-free survival. The plot compares the predicted survival probabilities with the observed survival outcomes, using calibration curves to assess model accuracy

**Fig. 5:**
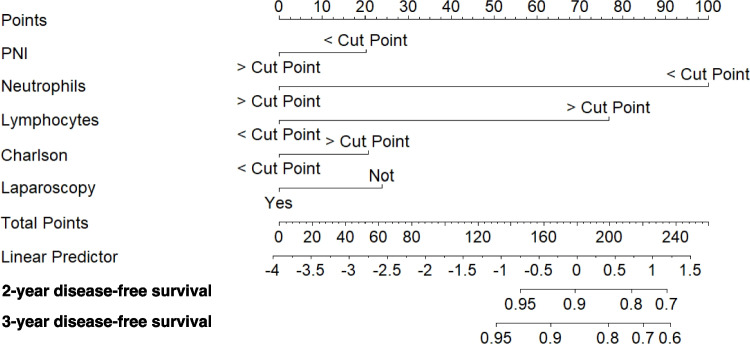
Nomogram of the Cox regression-based model for three-year disease-free survival. The nomogram visually represents the individual contribution of each variable to the predicted probability of survival at three years, allowing for personalized risk assessment in clinical practice. (PNI: Prognostic Nutritional Index)

**Fig. 6:**
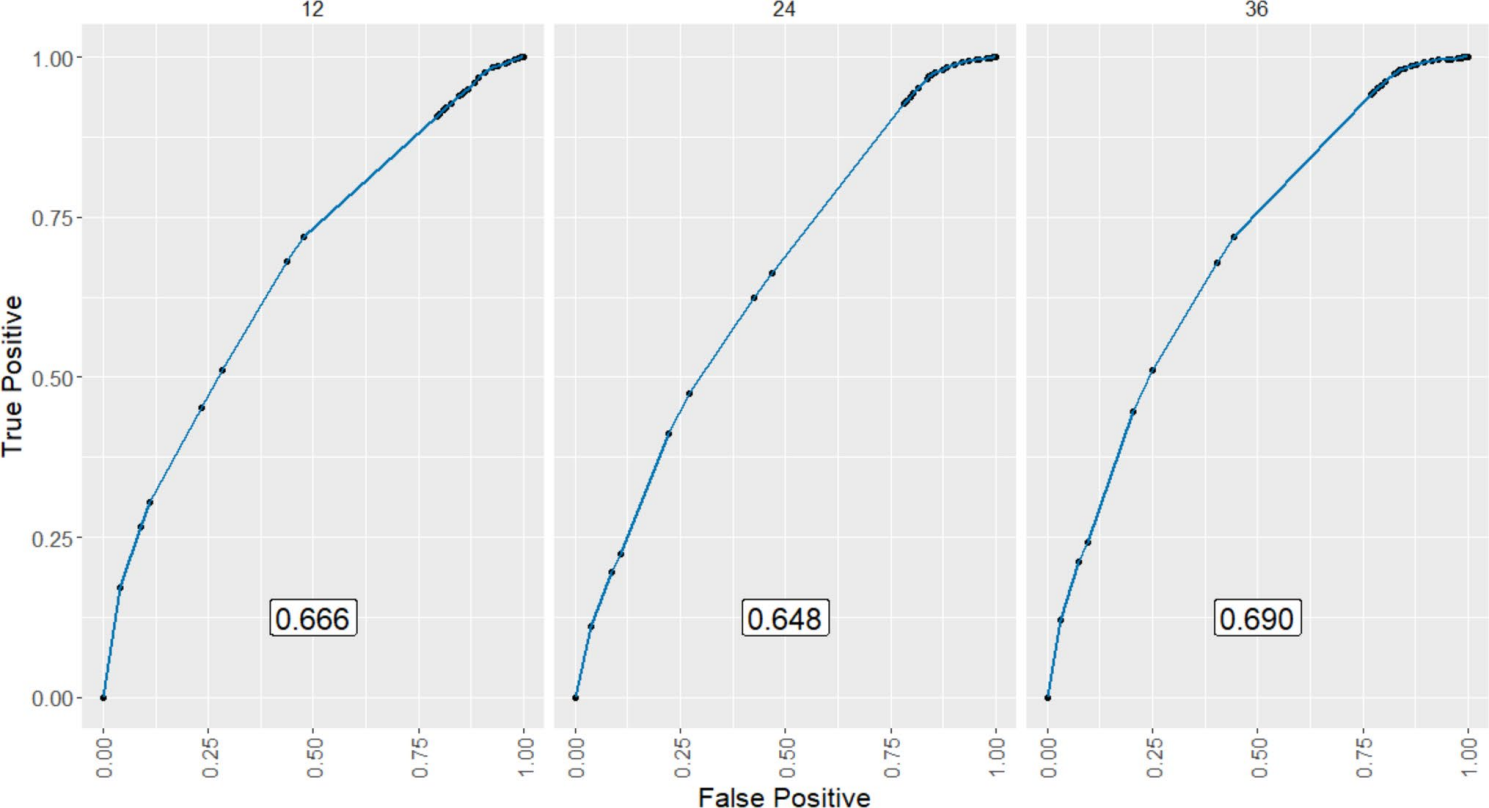
Receiver Operating Characteristic (ROC) curves for three-year disease-free survival in colorectal cancer. ROC curves were generated for each postoperative year to evaluate the model’s predictive performance at different time points

Multivariable Cox regression models for 3-year DFS and CSS were developed using the training dataset (Fig. 2). Independent prognostic factors for 3-year DFS included laparoscopic approach (*p* = *0.006*, VIF: 1.018), cut-off values for PNI (*p* = *0.019*, VIF: 1.115), neutrophils (*p* = *0.002*, VIF: 1.004), lymphocytes (*p* = *0.004*, VIF: 1.004), and Charlson comorbidity index (*p* = *0.018*, VIF: 1.11) [Table 4]. No severe multicollinearity was detected. Hazard ratios were computed for each independent prognostic variable of the model and variable importance was graphically represented (Fig. 3). Analysis of variance (ANOVA) revealed that neutrophil, lymphocyte, and laparoscopic approach cut-off values contributed most significantly to explain the variance of the 3-year recurrence model. The concordance index was 66.9% (SE = 0.022). Model validation was performed using sample splitting and bootstrap techniques, and calibration was assessed in the testing group (Fig. 4). A 3-year DFS nomogram was developed (Fig. 5), and the ROC curve analysis for the model showed AUC values of 66.6%, 64.8%, and 69% for years 1, 2, and 3, respectively (Fig. 6).

**Table 4:** Independent prognostic variables for 3-year disease-free survival. Cox regression model

	3-year disease-free survival			
	Coef	SE	VIF	*p*-value
PNI*	−0.449	0.188	1.115	0.019
Neutrophils*	−2.178	0.714	1.004	0.002
Lymphocytes*	1.668	0.586	1.004	0.004
Charlson comorbidity index*	0.449	0.190	1.110	0.018
Laparoscopic approach	−0.519	0.189	1.018	0.006

*Cut-off values of each variable were used for the analysis

**Fig. 7:**
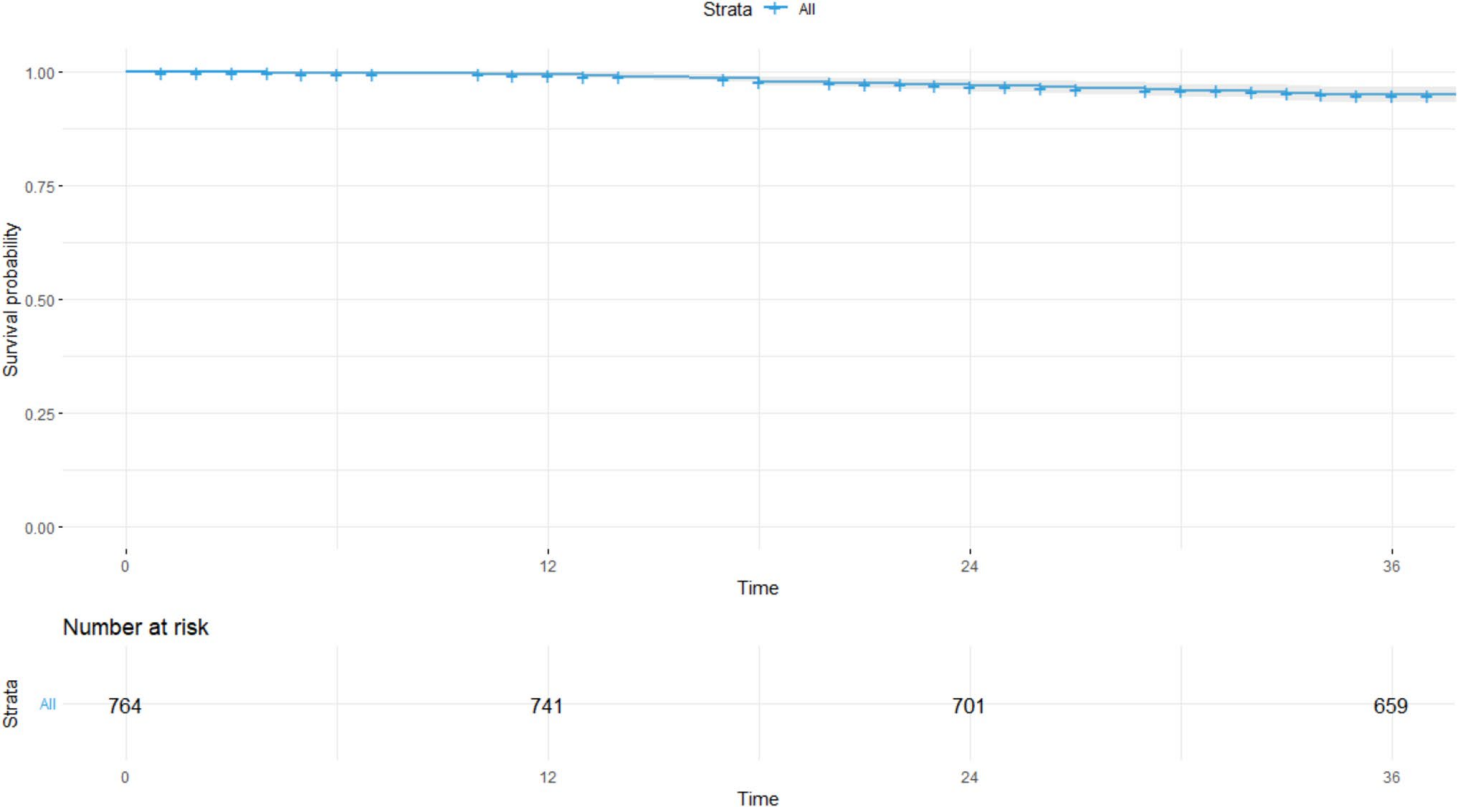
Kaplan–Meier curves showing 3-year cancer-specific survival probabilities based on the Cox regression model

**Fig. 8:**
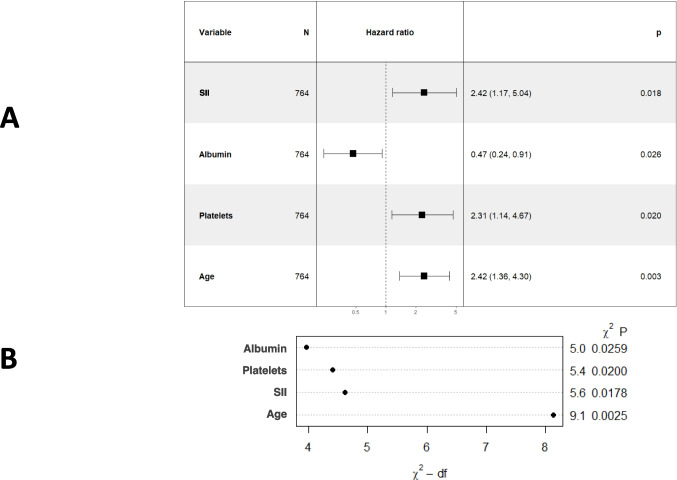
**A** Forest plot of the Cox regression-based model for 3-year cancer-specific survival. The plot displays the relative risks and confidence intervals for each variable included in the model, illustrating their impact on patient cancer-specific survival at three years. **B** Graphical representation of the importance of each variable in the Cox regression model for 3-year cancer-specific survival. This visualization highlights the contribution of each prognostic factor to the cancer-specific survival prediction. (SII: Systemic Immune-inflammation Index)

**Fig. 9:**
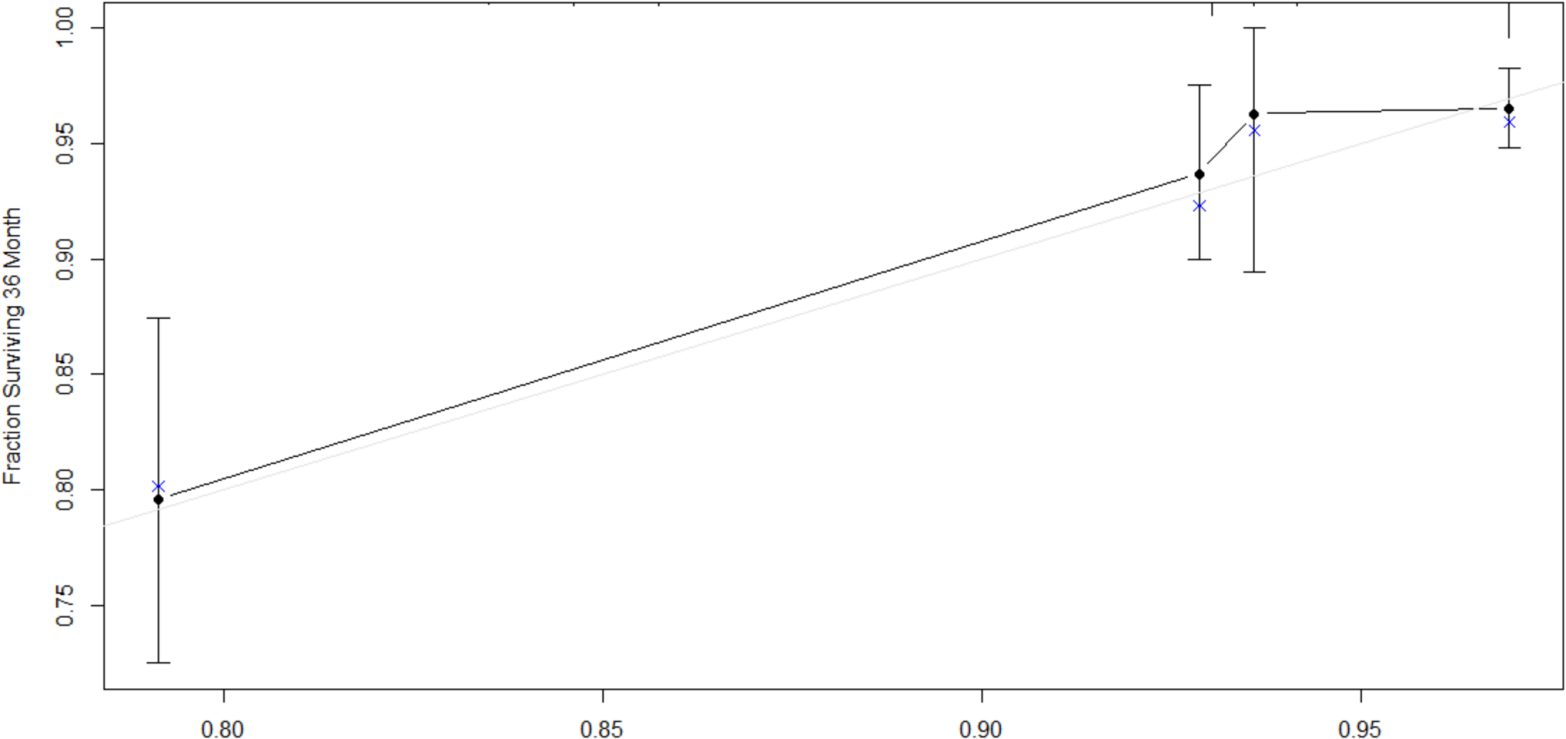
Calibration plot of the Cox regression-based model for 3-year cancer-specific survival. The plot compares the predicted cancer-specific survival probabilities with the observed outcomes, assessing the accuracy of the model’s predictions at three years

**Fig. 10:**
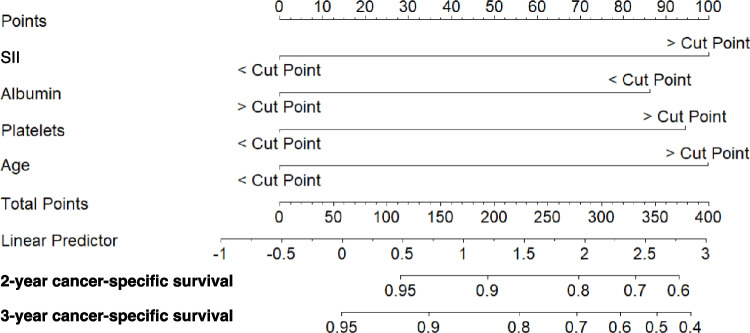
Nomogram of the Cox regression-based model for 3-year cancer-specific survival. This nomogram visually represents the contribution of each prognostic variable to the prediction of cancer-specific survival at three years, facilitating individualized survival risk assessment for patients. (SII: Systemic Immune-inflammation Index)

**Fig. 11:**
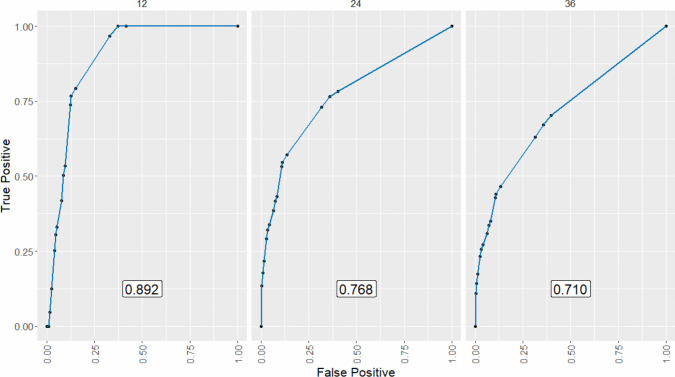
Receiver Operating Characteristic (ROC) curves for 3-year cancer-specific survival in colorectal cancer. ROC curves were generated for each postoperative year to assess the model’s predictive accuracy for cancer-specific survival at different time points

For 3-year CSS, the training group was also used to create a Cox regression-based model (Fig. 7). This model identified age cut-off (*p* = *0.003,* VIF: 1.039), SII cut-off (*p* = *0.018,* VIF: 1.544), serum albumin (*p* = *0.026*, VIF: 1.297), and platelets (*p* = *0.02*, VIF: 1.433) as independent prognostic factors. In this case, the hazard ratio for each variable of the model was also calculated. Assessment of VIF for the model variables ruled out the presence of severe multicollinearity. Forest plot and graphical representation of variable importance were created (Fig. 8A). ANOVA found several cut-off values that explained the most variance in the 3-year CSS model. The most significant contributors to model variance were SII and platelet cut-off values, while albumin played a lesser role (Fig. 8B). The Harrell’s concordance index was 71.4% (SE: 0.039). Calibration and validation were performed as described for DFS (Fig. 9). A nomogram for 3-year CSS was created based on this model (Fig. 10), with AUC values of 89.2%, 76.8%, and 71% for years 1, 2, and 3, respectively (Fig. 11).

### Discussion

The main finding of this study was the association of several serum biomarkers obtained from preoperative peripheral blood examination with definitive tumour staging, CSS and DFS. Certain patient-dependent characteristics and other systemic immune-inflammatory markers were independently related with 3-year DFS and 3-year CSS. A prognostic model for 3-year DFS was created including laparoscopic approach, cut-off values for PNI, neutrophils, lymphocytes, and Charlson comorbidity index, with an AUC of 69%. Similarly, a prognostic model for 3-year CSS was generated including age cut-off, SII cut-off, serum albumin, and platelets counts with an AUC of 71%.

Accumulating evidence from preclinical studies has demonstrated that high levels of neutrophils, platelets, monocytes or C-reactive protein promote tumour progression through the secretion of cytokines and chemokines, while lymphocytes and serum albumin have an anti-tumour effect, stimulating a cytotoxic immune response to cancer. Therefore, the combination of these parameters in the form of ratios, such as NLR, PLR, LMR, MLR, or SII, reflect the balance between the pro-tumour inflammatory state and anti-tumour immune status, contributing to the prediction of CRC prognosis [5, 7, 19].

The correlation between preoperative elevated NLR and poor survival outcomes in CRC was first reported by Walsh et al*.* [20]. However, several studies failed to find robust evidence of this ratio as an independent prognostic factor for DFS in resectable CRC [10, 21, 22]. Similarly, no association between NLR and DFS or CSS was observed in our study. Nonetheless, lymphocyte count itself was independently associated with DFS. Peripheral blood lymphocytes are involved in the elimination of circulating tumour cells through the cytotoxic T-cell immune response, playing a pivotal role in avoiding tumour progression. In previous research, high preoperative lymphocyte count was also an independent favourable prognostic factor for some CRC patient subsets [19, 23]. Conversely, neutrophils may indicate acute and chronic inflammation, facilitating tumour development and progression. Our results support that high serum neutrophil count itself was independently related to worse DFS. Similarly, previous studies found that elevated neutrophil count was also independently associated with worse survival outcomes in metastatic CRC patients with RAS wild-type mutations [5, 24].

Thrombocytosis is often observed in the inflammatory response. Platelets have an important function in tumorigenesis by facilitating angiogenesis and promoting tumour growth and invasion. In previous research, elevated platelet count was independently related to poor CSS. Similarly, there is emerging evidence for PLR as a solid CRC prognostic factor independently correlated with survival, as it involves both an increase in platelets and a decrease in lymphocyte count [7, 11, 25, 26]. Our study revealed that platelet count itself was an independent prognostic factor for CSS in CRC patients. Interestingly, we could not confirm any relationship between PLR with CSS or DFS. Other studies have also found controversial outcomes in the role of platelet count in CRC prognosis. Wan et al*.* studied a cohort of 1,513 surgically resected CRC patients and demonstrated that preoperative platelet count was significantly associated with metastasis after surgery but not with locoregional recurrence [27]. In the recently published metanalysis led by Guo et al., high PLR levels were correlated with OS or DFS, but no association was found with poorer CSS [9]. More comprehensive biomarkers have been developed, such as the SII, which includes peripheral neutrophil, platelet, and lymphocyte counts. SII was first described by Hu et al. in 2014 to predict the prognosis of hepatocarcinoma [28]. Further research demonstrated that an elevated SII was correlated with poor survival rates in patients with CRC [12, 13, 29, 30]. In the present study, SII together with platelets were the independent predictive factors found to contribute most to CSS prognosis in the model, and SII was also associated with histological differentiation and TNM stage. A recent metanalysis made a similar finding that high SII levels were significantly correlated with poor tumour differentiation [12]. Overall, elevated levels of neutrophils and platelets, alongside low levels of lymphocytes reflect a strong inflammatory response with a weak immune status. Thus, SII may more accurately represent the inflammatory state and immune response than the other markers. These findings seem to indicate that the host immune response plays an essential function in tumour progression. As an application in clinical practice, preoperative identification of elevated SII higher than 3.57 might warrant closer surveillance of this patient subgroup, especially in those with poor tumour differentiation.

Another widely studied biomarker is serum albumin, which reflects not only inflammatory status (as an acute-phase protein which decreases in response to inflammation) but also nutritional status, and is strongly correlated with poor oncological outcomes. In addition, albumin also plays a role in anticancer functions and anti-tumour therapy [31, 32]. The relationship between serum albumin and lymphocyte count is characterized through the PNI, which was initially introduced in 1984 by Onodera et al. as a predictor of postoperative complications [33], and which following extensive research is currently a well-known CRC prognostic marker. A reduced PNI negatively affects prognosis. Several studies set in Japan showed similar outcomes to ours, supporting that low PNI is independently associated with poor survival rates in CRC patients undergoing curative resection, but is also consistently associated with aggressive clinicopathological features, including large tumour size and high TNM stage [14, 34–36]. In the present study, PNI and serum albumin were independent predictive factors of DFS and CSS, respectively, consistent with previous studies. However, both markers had only a limited influence on CRC prognosis in our setting.

A correlation has been also reported between CRC prognosis and C-reactive protein as a nonspecific systemic inflammatory marker [37]. However, we chose not to include this serum marker in the analysis as it is not routinely examined as part of the preoperative work-up in CRC staging in our institution.

The high variability in cut-off values of biomarkers in previous research precludes drawing firm conclusions or establishing action protocols. This heterogeneity may owe to several factors dependent on the immune or genetic status of each patient, but the disparity of cut-off points used in different studies makes it impossible to establish useful reference values for clinical practice. Future research should be addressed at pinpointing thresholds for each parameter. In our work, the cut-off thresholds were calculated specifically for the study sample, providing values that are highly accurate, but not easily generalisable to other settings.

Individual inflammatory markers, though valuable, often exhibit limited predictive power when used in isolation. This may lead to biases and inconsistencies in prognostic evaluations. By integrating multiple biomarkers with patient-specific factors in this study, we significantly enhanced the precision of prognostic models, reflecting the multifactorial nature of cancer biology. A stronger association with CSS or DFS can be found if patient-dependent variables such as age or comorbidities are included. ROC analysis indicated high predictive value of the model for CSS when including age, SII, serum albumin level and platelet count, suggesting that immune response to the tumour and systemic inflammation are crucial in CRC patient survival. Similarly, Kudou et al. recently reported a novel index score based on sex, age, CEA level, C-reactive protein/albumin ratio, and PNI, which could predict CRC prognosis more accurately than the individual markers alone [38].

In the era of precision medicine, patient-specific management is required, so our prognostic nomograms have been designed for convenient and intuitive preoperative assessment of CRC patients in clinical practice. They provide individualised preoperative prognostic information identifying patients with higher risk of recurrence or death, for optimal selection of the best treatment and follow-up strategy for each patient.

Our study has several strengths. It was based on a relatively large and well-defined CRC patient cohort from a single centre. All patients were treated by the same colorectal surgeons with the same standardized surgical technique and the same postoperative management protocol, resulting in a homogeneous sample. Blood examinations were restricted to preoperative measurement to minimize other factors that could affect immune status, such as surgery, chemotherapy or radiation therapy. The outcomes were highly statistically significant in Cox regressions and subsequently validated with randomized sample splitting and bootstrap techniques. The models obtained exhibited a high predictive value, as it is shown in ROC curves.

Our AUC values for DFS (69%) and CSS (71%) are consistent with those reported in previous studies assessing inflammatory and nutritional biomarkers in colorectal cancer prognosis [12, 14]. While prognostic models based on systemic inflammation markers typically exhibit moderate discriminatory power, our results are in line with—or even superior to—those from similar analyses. These findings reinforce the relevance of inflammatory biomarkers in preoperative risk stratification and highlight their potential role in complementing existing prognostic tools.

Nevertheless, this study has several limitations that should be acknowledged. Although the sample size was relatively large, the study was conducted at a single centre and thus lacked external validation. Additionally, genetic, immunological, and demographic variability across different ethnic groups may limit the generalizability of these findings. Given that the models might perform differently in more diverse populations or surgical settings, the absence of external validation represents a significant limitation. Furthermore, while the AUC for the DFS model was statistically significant, its modest value suggests that it may not surpass other established prognostic tools commonly used in clinical practice.

Currently, there is no strong evidence to support altering patient management strategies based solely on preoperative immune or nutritional markers. Further prospective and interventional studies are necessary to determine whether modifications in the timing of surgery, the intensity of adjuvant therapy or surveillance protocols, based on these biomarkers will translate into improved outcomes.

To confirm the clinical utility of the proposed models, external validation is essential. A promising strategy would involve establishing multicentre collaborations with institutions of varying characteristics and including patients from diverse geographic and demographic backgrounds. Such an approach would help evaluate the robustness of these models in different surgical, demographic, and genetic contexts. Moreover, future studies could investigate incorporating emerging biomarkers such as cytokine profiles or immunogenetic markers, which may enhance the predictive performance of these models.

## Conclusion

In conclusion, this study highlights the prognostic value of routinely accessible preoperative biomarkers, such as SII, serum albumin, and PNI, in CRC. These markers not only reflect systemic inflammation and immune response but also provide actionable insights for clinical decision-making. This study confirms that inflammation-based parameters, when combined with clinical variables, serve as robust prognostic tools to accurately predict survival outcomes in patients undergoing curative resection for CRC. These findings underscore the critical role of systemic inflammation and immune response in tumour progression and survival. Moreover, immune markers were related to poor tumour differentiation and TNM staging. The advent of individualized prognostic models exemplifies the shift towards precision oncology, enabling tailored treatment and follow-up strategies. Future validation in multicentre cohorts will be essential to generalize these findings and further integrate them into clinical protocols.

## Data Availability

Data supporting the findings of this study are available from the corresponding author upon reasonable request.
